# The validity of dementia diagnoses in routinely collected electronic health records in the United Kingdom: A systematic review

**DOI:** 10.1002/pds.4669

**Published:** 2019-01-22

**Authors:** Luke A. McGuinness, Charlotte Warren‐Gash, Louisa R. Moorhouse, Sara L. Thomas

**Affiliations:** ^1^ Department of Population Health Sciences Bristol Medical School Bristol UK; ^2^ Faculty of Epidemiology and Population Health London School of Hygiene and Tropical Medicine London UK

**Keywords:** dementia, diagnosis, electronic health records, systematic review, United Kingdom, validity, pharmacoepidemiology

## Abstract

**Purpose:**

The purpose of the study is to assess the validity of codes or algorithms used to identify dementia in UK electronic health record (EHR) primary care and hospitalisation databases.

**Methods:**

Relevant studies were identified by searching the MEDLINE/EMBASE databases from inception to June 2018, hand‐searching reference lists, and consulting experts. The search strategy included synonyms for “Dementia”, “Europe”, and “EHR”. Studies were included if they validated dementia diagnoses in UK primary care or hospitalisation databases, irrespective of validation method used. The Quality Assessment for Diagnostic Accuracy Studies‐2 (QUADAS‐2) tool was used to assess risk of bias.

**Results:**

From 1469 unique records, 14 relevant studies were included. Thirteen validated individual diagnoses against a reference standard, reporting high estimates of validity. Most reported only the positive predictive value (PPV), with estimates ranging between 0.09 and 1.0 and 0.62 and 0.85 in primary care and hospitalisation databases, respectively. One study performed a rate comparison, indicating good generalisability of dementia diagnoses in The Health Improvement Network (THIN) database to the UK population. Studies were of low methodological quality. As studies were not comparable, no summary validity estimates were produced.

**Conclusion:**

While heterogenous across studies, reported validity estimates were generally high. However, the credibility of these estimates is limited by the methodological quality of studies, primarily resulting from insufficient blinding of researchers interpreting the reference test. Inadequate reporting, particularly of the specific codes validated, hindered comparison of estimates across studies. Future validation studies should make use of more robust reference tests, follow established reporting guidelines, and calculate all measures of validity.

**KEY POINTS**

- No prior review has systematically identified and appraised studies validating diagnostic codes/algorithms for dementia in UK electronic health records.
- Among the 14 included studies, the PPV of codes used to identify dementia ranged from 0.09 to 1.0 and 0.62 to 0.85 in primary and secondary care databases, respectively.
- Although reported estimates of validity were relatively high, all studies were at significant risk of bias.
- Few studies reported NPV, sensitivity, or specificity. Reporting PPV alone limits the generalisability of results to study periods with a different dementia prevalence.
- Future research is needed to develop and validate “best‐practice” dementia codes/algorithms for use in distinct EHR databases.

## 1 INTRODUCTION

The burden of dementia and its associated health and societal costs are projected to have major global impact as populations continue to age over the next few decades, despite the recent decline in age‐specific dementia incidence.^1^ With only one‐third of cases being due to potentially modifiable risk factors and in the absence of effective treatments,^2^ there is an urgent need for adequately powered studies seeking to identify dementia determinants. The recent widespread adoption of routine electronic health records (EHR) offers an opportunity to conduct powerful, efficient population‐based studies into dementia causes and outcomes. However, the advantages associated with the use of EHR data rely on the codes/algorithms used to identify dementia cases in these databases being valid.

Several large UK‐based EHR databases exist and are regularly used for research purposes, including the Clinical Practice Research Datalink (CPRD, formerly known as the GPRD), which as of 2013 covered ~7% of the UK population,^3^ and the Hospital Episode Statistics Admitted Patient Care (HES‐APC) database, which contains details on admissions to all National Health Service Trusts in England.^4^ Previous reviews have established the generally high validity of codes and algorithms for multiple conditions in a single database, such as the CPRD.^5^ However, no published systematic review has specifically examined the validity of codes/algorithms used to identify dementia cases across all UK EHR databases.

The objectives of this review were to systematically identify and appraise studies validating diagnostic codes/algorithms for dementia in UK primary care and hospitalisation electronic health records, summarise their results to identify best‐practice codes/algorithms for dementia in each distinct database, and to highlight gaps in the literature to inform future research.

## 2 METHODS

We performed a systematic review of studies validating dementia diagnoses in UK primary or secondary care electronic health records. This review was conducted in accordance with the guidelines of the Cochrane Collaboration Diagnostic Test Accuracy handbook^6^ and the PRISMA statement.^7^

### 2.1 Eligibility criteria

We sought to include primary studies that validated a code or algorithm used to identify dementia cases in a UK population‐based primary care or hospitalisation EHR database. Acceptable methods of validation included comparison against a reference standard (for example, GP questionnaire, case note review, external database, or disease‐specific registry) or a comparison of rates with external population‐based data. Case note review in this context refers to a thorough review of the entire patient record, including the free text fields of the EHR database and copies of hospital letters, when available. To be included, a study validating individual diagnoses against a reference standard must have reported at least one measure of validity, namely the positive predictive value (PPV), negative predictive value (NPV), sensitivity, or specificity, or the raw data to allow for its calculation. No restrictions were made relating to the UK database examined, the language or date of publication, or the number of validations performed. Conference abstracts were included, if eligible, provided the relevant details could be obtained from the authors.

We did not include validations of dementia diagnoses in death certificates or disease‐specific registries, as these were considered beyond the scope of this review. Studies validating only the date of diagnosis were excluded.

### 2.2 Search strategy

We searched MEDLINE and EMBASE from inception to 25 June 2018. Searches used a combination of free text and controlled vocabulary terms to identify studies conducted in UK EHR databases in which a diagnostic validation of dementia was reported. The search strategy included synonyms for “Dementia” AND “Europe” AND “EHR” (Appendix 1). We did not include synonyms for “Validity” as it had previously been shown that terms relating to the validation of diagnoses are often not mentioned in the searchable terms of a reference (the title, abstract, and controlled vocabulary), particularly when validation represented only a small aspect of a larger study.^5^, ^8^ We used synonyms for Europe in place of terms for the UK, as we had identified some records examining multiple European EHR sources that contained information relevant to UK databases, but were not captured by a search incorporating only UK terms. We also searched for synonyms of “Dementia” AND UK‐specific databases such as the CPRD.

Bibliographies of the CPRD,^9^ the Boston Collaborative Drug Surveillance Programme,^10^ and The Health Improvement Network (THIN)^11^ were hand‐searched to identify additional relevant articles. The bibliographies of systematic reviews of the validity of multiple diagnoses contained within UK EHR databases, identified both through consultation with subject matter experts and through the search strategy, were screened for potentially relevant articles. Finally, the reference lists of included studies were screened, while the articles that had cited included studies were examined using Google Scholar.

### 2.3 Study selection

The titles and abstracts of retrieved references were screened to identify studies that possibly examined UK EHR data. Only when it was clear that a study did not use EHR data (for example, the abstract listed the specific European countries/databases examined) was a reference excluded. The full texts of all remaining studies were then assessed for inclusion in the review. Eligibility assessment was performed by one reviewer (L.A.M.), with a second reviewer (L.R.M.) independently assessing a random sample of 30% of references at each stage to verify the screening process.

### 2.4 Data extraction

Data extraction was performed by one reviewer (L.A.M.) using a standardised data abstraction form and was reexamined by a second reviewer (L.R.M.) to ensure accuracy. When the relevant details were not adequately reported in the study's publication, the primary author was contacted. We extracted data on the study authors, date of publication, setting, study period, database, diagnostic codes (OXMIS, Read, International Classification of Diseases) used to define dementia, characteristics of the study population, validation method used, number and proportion of identified dementia cases that were validated and measures of validity reported (PPV, NPV, sensitivity, specificity), or the raw data to allow their calculation.

### 2.5 Quality assessment

The studies included in this review most closely resemble diagnostic accuracy studies. Based on this, we used the Quality Assessment of Diagnostic Accuracy Studies 2 (QUADAS‐2) tool,^12^ tailored to our review question, to assess the risk of bias and applicability of included studies which validated a dementia diagnosis using a reference standard. QUADAS‐2 consists of four domains (study design and patient selection, index test, reference standard, flow and timing), with each being assessed in terms of risk of bias and the first three in terms of applicability concerns. In the context of this review, the phrase “index test” refers to the codes/algorithms which were tested for their ability to accurately define a dementia diagnosis. Each study was initially assessed by one reviewer (L.A.M.), and the risk of bias judgements made was reevaluated by a second researcher (L.R.M.). Disagreements were resolved by discussion with a senior member of the review team (S.L.T.).

### 2.6 Analysis

For each included study which validated individual diagnoses against a reference standard, the PPV, NPV, sensitivity, and specificity of the code/algorithm examined were calculated when necessary from the raw data, along with 95% confidence intervals.^13^, ^14^ Stata Statistical Software Package version 14.0 (StataCorp LP, College Station, TX, USA) was used to present coupled forest plots of the PPV/NPV and sensitivity/specificity,^15^ stratified by setting (primary care vs hospitalisation), EHR database, and dementia type. Studies validating <10 dementia cases were excluded from the plots.

Due to a lack of comparable studies following stratification, summary estimates of validity could not be produced, and a formal assessment of heterogeneity and publication bias could not be performed. As an exploratory analysis, the heterogeneity between the PPV estimates within strata was examined using a *χ* ^2^ test.^13^

## 3 RESULTS

### 3.1 Literature search

**Figure 1:**
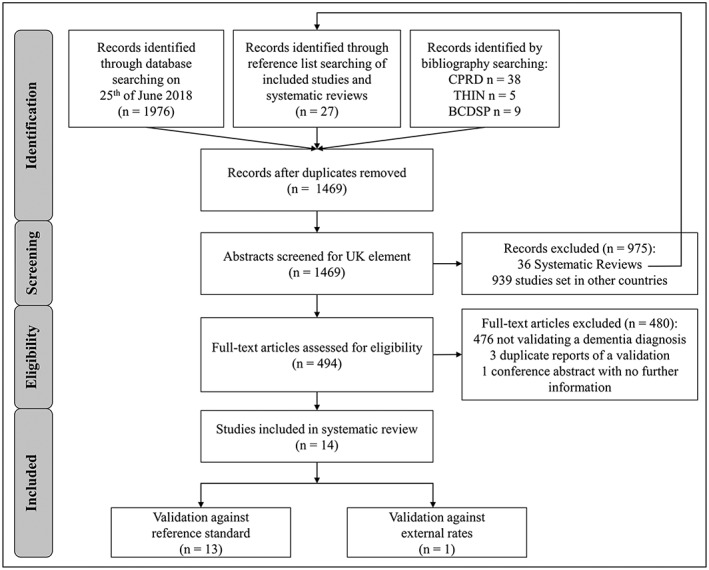
PRISMA flow chart

We identified 2055 records through the database search and bibliography screening (Figure 1). Following deduplication, the titles and abstracts of 1469 unique records were screened for inclusion in the review, of which 975 were determined not to be primary validation studies of a UK database. Fourteen of the 494 remaining studies were included after full‐text screening. Reasons for exclusion at this stage included lack of a dementia diagnoses validation (n = 476), duplicate reporting of a previous published validation (n = 3), and a conference abstract for which no further information could be obtained (n = 1).

### 3.2 Characteristics of included studies

Fourteen references met the inclusion criteria (Table 1),^16^, ^17^, ^18^, ^19^, ^20^, ^21^, ^22^, ^23^, ^24^, ^25^, ^26^, ^27^, ^28^, ^29^ of which 13 used reference standards to validate individual diagnoses of dementia within an EHR database. Half of the included studies had validation of diagnoses as the primary aim of the study (n = 7). Seven studies were UK‐wide, four examined Scottish EHR databases, and three examined an English database. Ten studies examined primary care databases, with the CPRD being the most commonly validated (n = 6), while four examined hospitalisation data. Half (n = 7) of the included studies specified the exact codes/algorithms that they sought to validate (Table 2).

**Table 1:** Summary of characteristics of studies included in the review (stratified by setting, database, and dementia type)

Study	Country	Setting	Study Design	Study Period	Database	Coding System	Characteristics of Population to Which Index Test Was Applied			Diagnosis Validated	Validation Method
							Description	Age	Sex		
Walker, 2018^16^	England	Primary care	Prescribing trend analysis	1 January 1987‐31 December 2016	CPRD	READ	Patients with at least 12 consecutive months of records classified as “acceptable” by the CPRD from an “up to standard” practice.	>40	M/F	AD + dementia	External database comparison (HES + ONS)
Whitelaw, 1996^17^	Scotland	Primary care	Validation	April 1992	GPASS	READ	Random sample of 250 patients from 41 practices (those having more than 50% of patients with a clinical Read code) that volunteered to take part in the study	45‐64	M/F	Dementia	Case note review
Seshadri, 2001^18^	UK	Primary care	Population‐based nested case‐control	1 January 1990‐31 October 1998	GPRD	READ	Women in the population who had received ≥1 prescription for a systemic (oral or transdermal) oestrogen preparation during study period.	>40	F	AD	Case note review
Imfeld, 2012^19^	UK	Primary care	Population‐based nested case‐control	January 1998‐November 2008	GPRD	READ	Patients with a diagnosis of AD during study period	>65	M/F	AD	GP confirmation^b^
Dunn, 2005^20^ (a)^a^	UK	Primary care	Population‐based nested case‐control	1 January 1992‐1 January 2002	GPRD	READ	Patients included in GPRD during study period	>60	M/F	Dementia	GP confirmation^b^
Dunn, 2005^21^ (b)^a^	UK	Primary care	Population‐based nested case‐control	1 January 1992‐1 January 2002	GPRD	READ	Patients included in GPRD during study period	>60	M/F	Dementia	GP confirmation^b^
Imfeld, 2013^22^	UK	Primary care	Population‐based nested case‐control	January 1998‐November 2009	GPRD	READ	Patients with a diagnosis of VaD during study period	>65	M/F	VaD	GP confirmation^b^
Heath, 2015^23^	Scotland	Primary care	Population‐based cross‐sectional study	March 2007	SPICE	READ	Patients with code for dementia at time of study from 314 general practices in Scotland that agreed to take part in the study.	40‐64	M/F	Dementia	Case note review
Blak, 2011^24^	UK	Primary care	Validation	2006‐2007	THIN	READ	Patients included in THIN	All	M/F	Dementia	External rate comparison
Van Staa, 1994^25^	UK	Primary care	Validation	30 April 1990‐31 July 1992	VAMP	ICD‐9	All persons who received during study period at least one prescription for glibenclamide, gliclazide, chlorpropamide, glipizide, or tolbutamide.	>20	M/F	Dementia	GP confirmation and review of discharge letters
Sommerlad, 2018^26^	England	Hospitalisation	Validation	1 January 2008‐1 March 2016	HES	ICD‐10	Patients with a diagnosis of AD during study period	>65	M/F	Dementia	External database comparison (CRIS)
Brown, 2016^27^	England	Hospitalisation	Validation	1 April 1997‐.31 April 2011	HES	ICD‐10	Women recruited from England into the million women study	55‐79	F	Dementia	External database comparison (CPRD) + GP confirmation and review of discharge letters
Soo, 2014^28^	Scotland	Hospitalisation	Validation	1 January 2003‐1 June 2003	SMR01	ICD‐10	Patients included in the GLOMMS‐I cohort (ie, identified as having moderate/severe chronic kidney disease during study period).	>15	M/F	Dementia	Case note review
Ryan, 1994^29^	Scotland	Hospitalisation	Validation	1968‐1987	SMR01/SMR04	ICD‐9^c^	Admissions to Scottish hospitals in the Lothian region during study period	N/A	M/F	Dementia	Case note review

Abbreviations: AD, Alzheimer's disease; CPRD, Clinical Practice Research Datalink; GLOMMS‐I, Grampian Laboratory Outcomes Mortality and Morbidity Study‐1; GP, general practitioner; GPASS, General Practice Administration System for Scotland; GPRD, General Practice Research Database; HES, Hospital Episode Statistics; ICD‐9, International Classification of Disease (9th Edition); ICD‐10, International Classification of Disease (10th Edition); QOF, Quality Outcome Framework; SMR01, Scottish Morbidity Records (General); SMR04, Scottish Morbidity Records (Psychiatric); THIN, The Health Improvement Network; VaD, Vascular Dementia; VAMP, Value Added Medical Products.

a Separate studies were performed by same author in same year. Study (a) examined whether lithium therapy could be used to prevent the onset of dementia, while study (b) examined the association of dementia with infection. While many aspects of the two studies appear quite similar, the discrepancy between the number of patients validated in each (Table 3) indicates that they should be treated as separate studies.

b For these studies, the method of GP confirmation is poorly described. Each study reported sending GPs a questionnaire to confirm the diagnosis of dementia (or its subtypes) but provided no details on the content of the questionnaire and did not mention requesting supporting materials such as hospital discharge letters.

c Harmonisation between ICD‐8 and ICD‐9 occurred for the period 1968 to 1979.

**Table 2:** Summary of diagnoses validated in the seven studies which reported the specific codes validated

Study	Coding System	Diagnosis Validated	Specific Codes/Algorithms Representing the Diagnosis	Notes
Walker, 2018	READ	Probable AD	Any of: Eu00.00, Eu00000, Eu00011, Eu00012, Eu00013, Eu00100, Eu00111, Eu00112, Eu00113, Eu00200, Eu00z00, Eu00z11, F110.00, F110000, F110100, Fyu3000	Patients may also have codes on the “possible AD” list and codes representing a prescription for a drug used exclusively in the treatment of dementia, detailed in BNF section 4.11, excluding idebenone.
		Possible AD	Any of: E00..00, E00..11, E00..12, E000.00, E001.00, E001000, E001100, E001200, E001300, E001z00, E002.00, E002000, E002100, E002z00, E003.00, E00z.00, Eu02z11, Eu02z12, Eu02z14, Eu02z15, Eu02z16, Eu05700, F11z.11	Patients may also have codes on the “possible AD” list and codes representing a prescription for a drug used exclusively in the treatment of dementia, detailed in BNF section 4.11, excluding idebenone.
		Non‐AD And mixed dementia	Any of: Eu02z13, Eu02z00, Eu02.00, Eu02y00, 6AB..00, E041.00, 9hD1.00, 9hD0.00, E00y.00, F112.00, 8BPa.00, E02y100, Eu01111, Eu02000, Eu02100, Eu02200, Eu02300, Eu02400, Eu02500, Eu04100, F111.00, F116.00, F11x200, E004.00, E004.11, E004000, E004100, E004200, E004300, E004z00, Eu01.00, Eu01.11, Eu01000, Eu01100, Eu01200, Eu01300, Eu01y00, Eu01z00 or any code representing a prescription for a drug used exclusively in the treatment of dementia, detailed in BNF section 4.11, excluding Idebenone	Patients may also have codes on the “probable AD” and “possible AD” lists, but due to the presence of a non‐AD specific code, they do not meet the criteria for these classifications.
Heath, 2015	READ	Dementia	Any of: 66 h, 6AB.., E00.., E000., E001., E0010, E0011, E0012, E0013, E001z, E002., E0020, E0021, E002z, E003., E004., E0040, E0041, E0042, E0043, E004z, E00y., E00z., E041., Eu00., Eu000, Eu001, Eu002, Eu00z, Eu01., Eu010, Eu011, Eu012, Eu013, Eu01y, Eu01z, Eu02., Eu020, Eu021, Eu022, Eu023, Eu024, Eu025, Eu02y, Eu02z, F110., F1100, F1101, F111., F112., F116., Fyu30(in addition to codes representing a prescription for a drug used exclusively in the treatment of dementia, detailed in BNF section 4.11)	
Blak, 2011	READ	Dementia	Any of: E11.%, F212., F21Z, F371^a^	
Sommerland, 2018	ICD‐10	Dementia	Any of: F00x‐F03x, G30x, G31.0, G31.8	
Brown, 2016	ICD‐10	Dementia	Any of: E512, F00, F01, F02, F03, F10.6, F10.7, G30, or G31.0.	
Soo, 2014	ICD‐10	Dementia	Any of: F01, F00, F03, F02, F05.1, G30, G31.1	
Ryan, 1994	ICD‐9^b^	Dementia	290	

Abbreviations: AD, Alzheimer's disease; ICD‐9, International Classification of Disease (9th Edition); ICD‐10, International Classification of Disease (10th Edition).

a The “%” symbol represents a wildcard for READ codes (ie, a search using E11.% would identify E11 and all codes nested under it).

b Harmonisation between ICD‐8 and ICD‐9 occurred for the period 1968 to 1979.

### 3.3 Validity of dementia diagnosis

The 13 studies which used a reference standard reported 55 individual validity estimates (Table 3). Five studies only validated patients with a positive index test, reporting PPV as the sole validity measure. Three studies reported more than one PPV within the same paper, with two separate algorithms being compared against the same reference standard in the first,^18^ the same code/algorithm being compared against two separate reference standards in the second,^27^ and three separate algorithms being compared against two distinct reference standards in the third.^16^

**Table 3:** Summary of results of studies included in the review (stratified by setting, database, and dementia type)

Study	Diagnosis Validated	Validation Method	Index Dementia Positive			Index Dementia Negative			TP	FP	TN	FN	PPV	NPV	Sensitivity	Specificity
			Number Identified in Database	Number Chosen for Validation	Number with Available Data (%)	Number Identified in Database	Number Chosen for Validation	Number with Available Data (%)								
Walker, 2018^16^	Probable AD	Comparison against HES	8069	8069	8069 (100%)	21 293	21 293	21293 (100%)	2974	5095	19260	2033	0.37 (0.36‐0.38)	0.90 (0.90‐0.91)	0.59 (0.58‐0.61)	0.79 (0.79‐0.80)
		Comparison against ONS	8069	8069	8069 (100%)	21 293	21 293	21293 (100%)	1220	6849	20650	643	0.15 (0.14‐0.16)	0.97 (0.97‐0.97)	0.65 (0.63‐0.68)	0.75 (0.75‐0.76)
	Possible AD	Comparison against HES	8259	8259	8259 (100%)	21 103	21 103	21103 (100%)	2410	5849	17052	4051	0.29 (0.28‐0.30)	0.81 (0.80‐0.81)	0.37 (0.36‐0.38)	0.74 (0.740.75)
		Comparison against ONS	8259	8259	8259 (100%)	21 103	21 103	21103 (100%)	1711	6548	18062	3041	0.21 (0.200.22)	0.86 (0.850.86)	0.36 (0.35‐0.37)	0.73 (0.730.74)
	Non‐AD and mixed dementia	Comparison against HES	13 034	13 034	13 034 (100%)	16 328	16 328	16328 (100%)	4444	8590	14566	1762	0.34 (0.33‐0.35)	0.89 (0.89‐0.90)	0.72 (0.70‐0.73)	0.63 (0.62‐0.64)
		Comparison against ONS	13 034	13 034	13034 (100%)	16 328	16 328	16328 (100%)	1171	11863	16043	285	0.09 (0.09‐0.09)	0.98 (0.98‐0.98)	0.80 (0.78‐0.82)	0.58 (0.57‐0.58)
Whitelaw, 1996^17^, ^a^	Dementia	Case note review	?	?	?	?	?	?	‐	‐	‐	‐	1	‐	0	‐
Seshadri, 2001^18^	AD	Case note review	128	128	128 (100%)	‐	‐	‐	62	66	‐	‐	0.48 (0.4‐0.57)	‐	‐	‐
	AD	Case note review	51	51	51 (100%)	‐	‐	‐	43	8	‐	‐	0.84 (0.72‐0.92)	‐	‐	‐
Imfeld, 2012^19^	AD	GP confirmation	7086	60	60 (100%)	‐	‐	‐	47	13	‐	‐	0.79 (0.66‐0.87)	‐	‐	‐
Dunn, 2005 20 (a)^b^	Dementia	GP confirmation	9954	150	150 (100%)	9347	50	50 (100%)	150	0	50	0	1 (0.98‐1)	1 (0.93‐1)	1 (0.98‐1)	1 (0.93‐1)
Dunn, 2005^21^ (b)^b^	Dementia	GP confirmation	9954	100	95 (95%)	9374	50	55 (110%)^c^	79	16	55	0	0.83 (0.74‐0.89)	1 (0.93‐1)	1 (0.95‐1)	0.78 (0.66‐0.86)
Imfeld, 2013^22^	VaD	GP confirmation	4438	60	60 (100%)	‐	‐	‐	44	16	‐	‐	0.73 (0.61‐0.83)	‐	‐	‐
Heath, 2015^23^	Dementia	Case note review	15	15	15	‐	‐	‐	15	0	‐	‐	1 (0.80‐1)	‐	‐	‐
Van Staa, 1994^25^	Dementia	GP confirmation	NS	NS	12 (−%)	‐	‐	‐	12	0	‐		1 (0.76‐1)	‐	‐	‐
	Dementia	GP confirmation	NS	NS	9 (−%)	‐	‐	‐	7	‐	‐	2	‐	‐	0.78 (0.45‐0.94)	‐
Sommerland, 2018^26^	Dementia	Comparison against CRIS	8069	8069	8069	13 318	13 318	13318	6429	1640	12 094	1817	0.80 (0.79‐0.81)	0.87 (0.86‐0.88)	0.78 (0.77‐0.79)	0.88 (0.88‐0.89)
Brown, 2016^27^	Dementia	Comparison against CPRD	340	340	340 (100%)	‐	‐	‐	288	52	‐	‐	0.85 (0.80‐0.88)	‐	‐	‐
	Dementia	GP confirmation	NS	333	244 (73%)	NS	1004	866 (86%)	208	36	865	1	0.85 (0.80‐0.89)	1 (0.99‐1)	1 (0.97‐1)	0.965‐0.97)
Ryan, 1994^29^	Dementia	Case note review	1988	200	146 (73%)	‐	‐	‐	123	23	‐	‐	0.84 (0.77‐0.89)	‐	‐	‐
Soo, 2014^28^, ^d^	Dementia	Case note review	91	91	91 (100%)	3128	3128	3128 (100%)	56	35	2997	131	0.62 (0.51‐0.71)	0.96 (0.95‐0.96)	0.3 (0.24‐0.37)	0.99 (0.98‐0.99)

Abbreviations: AD, Alzheimer's disease; CRIS, Clinical Record Interactive Search at South London and Maudsley; FN, false negative; FP, false positive; HES, Hospital Episode Statistics; ONS, Office for National Statistics; NS, not stated; TN, true negative; TP, true positive; VaD, vascular dementia.

a Primary analysis unclear, reported validity estimates displayed for reference.

b Separate studies performed by same author in same year.

c More cases were validated than were specified in the study's methods.

d 95% CI was only reported by this study. For the remainder, the CIs were calculated using the Wilson method.

**Figure 2:**
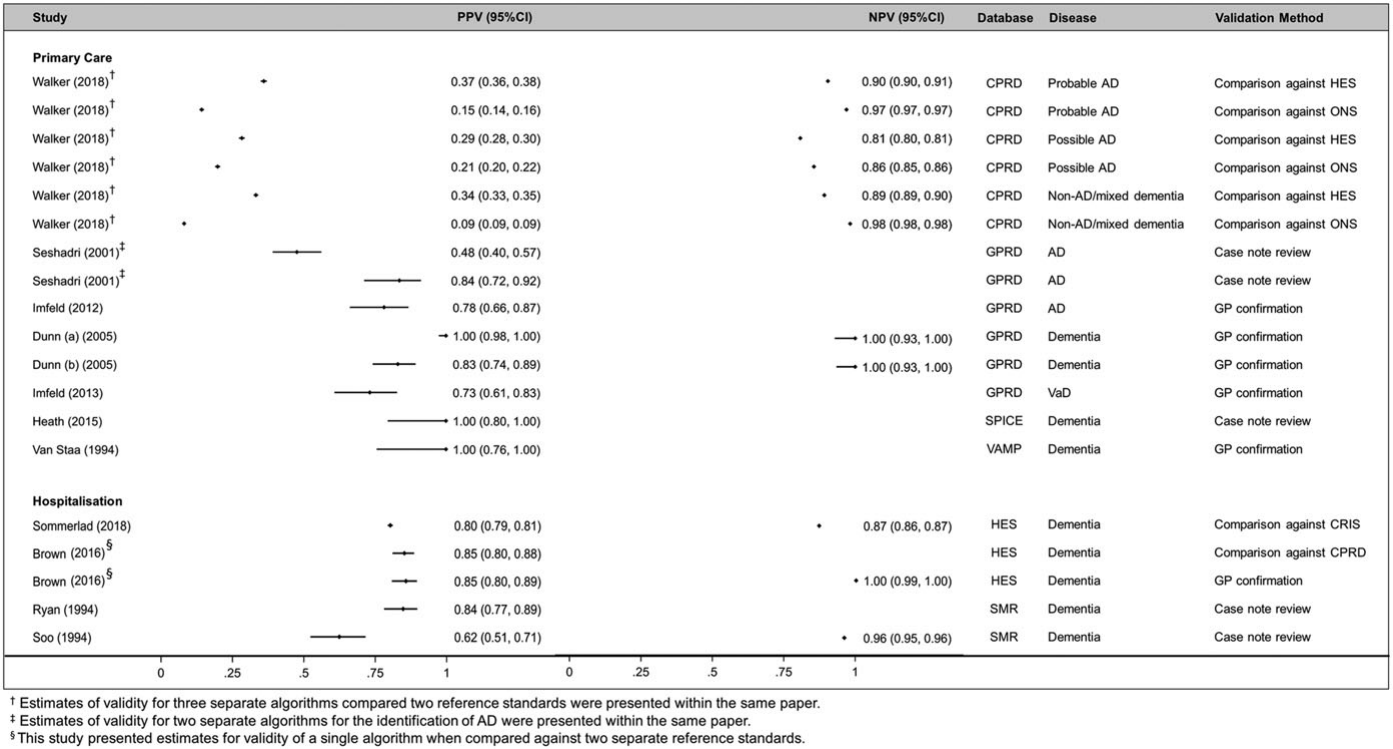
Coupled forest plot of PPV/NPV estimates (stratified by setting, database, and dementia subtype)

**Figure 3:**
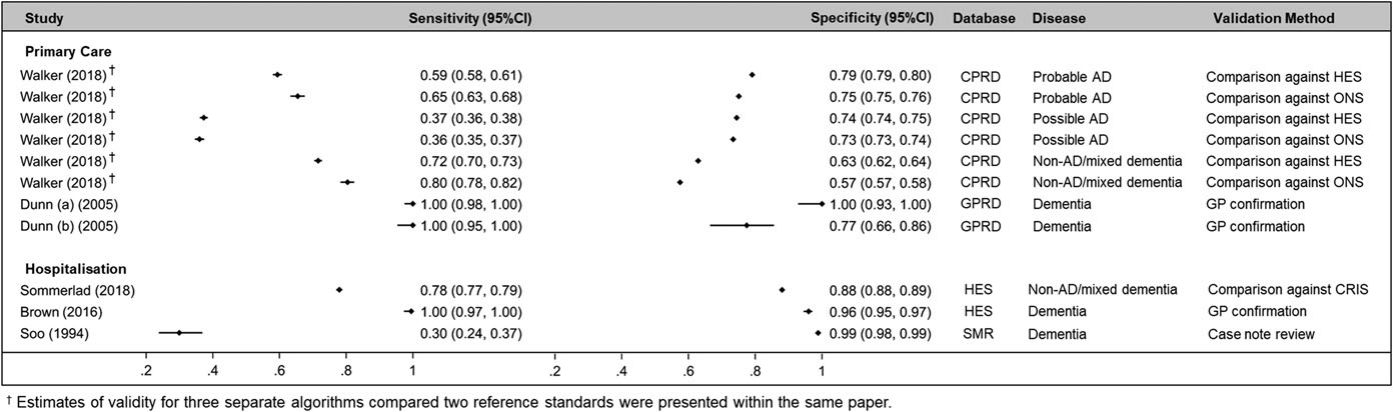
Coupled forest plot of sensitivity/specificity estimates (stratified by setting, database, and dementia subtype)

The estimates for each measure of validity, along with their 95% confidence intervals, are summarised in Figures 2 and 3. The PPV estimates reported varied between 0.09 and 1.0 in primary care databases and between 0.62 and 0.85 in hospitalisation databases. One study (Whitelaw et al^17^) was included in Table 1 but excluded from the forest plots due to both data scarcity (only three validations performed) and suboptimal reporting making it difficult to determine how the primary analysis was conducted.

One study conducted a rate comparison to validate a dementia code/algorithm, using the same set of codes to calculate the crude prevalence rates from both the THIN database and national Quality and Outcomes Framework (QOF) data for 2006 to 2007. While not directly providing validity measures, the estimated prevalence in the THIN database was slightly higher but comparable to that of the QOF data (0.47% ± 0.03% vs 0.42%, respectively).^24^

### 3.4 Risk of bias assessment

**Figure 4:**
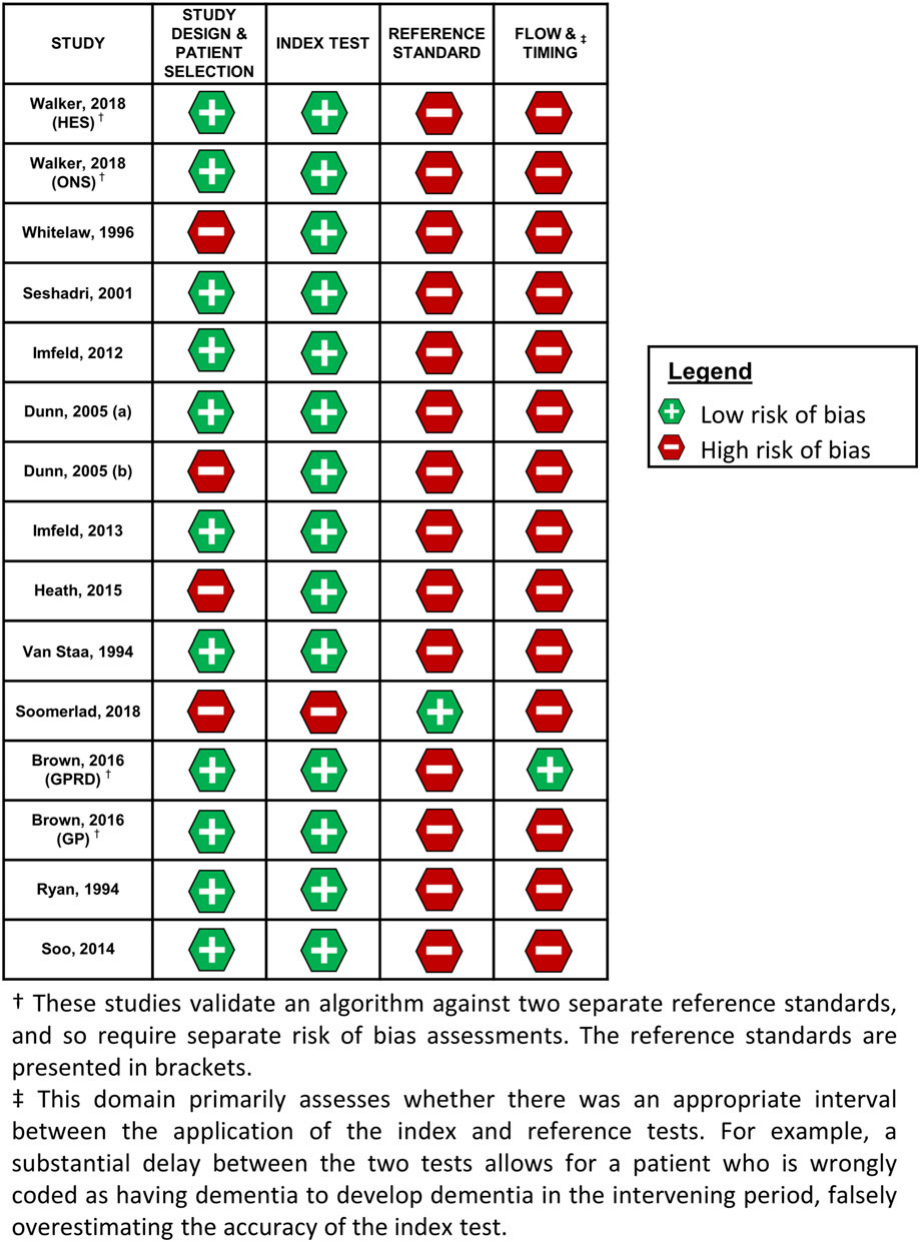
Risk of bias in each included study across the four QUADAS‐2 domains (study design and patient selection, index test, reference standard, and flow and timing) [Colour figure can be viewed at http://wileyonlinelibrary.com]

In Figure 4, we present the summary of our risk of bias assessment of included studies using the QUADAS‐2 tool. In general, studies had low internal validity, with all having a high risk of bias in at least one domain. For the study design and patient selection domain, the method used to select the subset of cases for validation was often not clearly stated. Application of the index test was at low risk of bias for almost all studies. Conversely, most studies were at high risk of bias related to application of the reference test, as few studies sufficiently blinded researchers to the results of the index test when interpreting the reference test (Appendix 2).

Assessment of the flow and timing domain of the QUADAS‐2 tool proved particularly challenging, as for most studies, the time between application of the reference and index tests was not clearly stated, allowing for potential delayed verification bias.

All studies had low concerns regarding their applicability to the review question. This was expected due to the necessarily broad review question.

### 3.5 Heterogeneity

There was evidence for heterogeneity between the PPV estimates for Alzheimer's (n = 7; *χ* ^2^ test for homogeneity of proportions = 1400; *P* < 0.001) and dementia (n = 4; *χ* ^2^ test = 3100; *P* < 0.001) in the CPRD, dementia in HES (n = 3; *χ* ^2^ test = 9.44; *P* = 0.009), and dementia in SMR (n = 2; *χ* ^2^ test = 15.64; *P* < 0.001). No evidence for heterogeneity was observed between PPV estimates for dementia in HES (*χ* ^2^ test = 0.032, *P* = 0.857).

## 4 DISCUSSION

### 4.1 Summary

The studies identified in this review included a range of settings, EHR databases, and dementia subtypes and validated varying codes/algorithms used to identify dementia. Validity estimates were generally high, though were heterogeneous across studies. Generally, poor reporting made it difficult to interpret and compare estimates of validity across studies, particularly as not all studies reported the specific code/algorithm validated.

### 4.2 Validity estimates

Several included studies which compared individual diagnoses to a reference standard reported solely the PPV of the code/algorithm they examined, a similar finding to previous reviews of EHR validation studies.^5^ PPV estimates were generally high, although they varied substantially between studies, ranging from 0.09 to 1.0 in primary care data and 0.62 to 0.85 in hospitalisation data. While the PPV is a useful measure, it is dependent on the prevalence of the dementia in the population at the time of study, and so its generalisability to different study periods is limited. This limitation is particularly important in the absence of the other measures of validity.

There is no universal threshold above which a PPV estimate is deemed acceptable because the relative importance of a test's validity is related to the specific consequences of misclassification. While generally high, some of the reported PPVs are likely suboptimal for the accurate classification of a patient's dementia status in future research, potentially introducing strong bias into a study performed using the corresponding code/algorithm.

Eight studies reported additional measures of validity. Like the PPV, estimates varied over a wide range of values but were generally high. An important exception is the low sensitivity (0.3, 95% CI: 0.24‐0.37) of an algorithm to identify dementia in the Scottish Mortality Records (SMR) database. One possible explanation for this is the restricted population (patients identified as having moderate/severe chronic kidney disease during study period).

The within‐strata heterogeneity of PPV estimates may primarily be explained by the differing time periods examined by individual studies. However, the contrast between the PPV estimates reported in two studies by Dunn et al merits further discussion. Although these studies were performed by the same author and are identical in terms of reported study characteristics, both were included in this review as the very different PPVs (0.83 vs 1) suggest that a different algorithm was used in each.^20^, ^21^ However, the specific codes validated were not reported so it remains possible that these studies had overlapping validation cohorts. Further, it is important to note that this heterogeneity assessment was exploratory and susceptible to bias as the *χ* ^2^ test does not account for the paired nature of validity measures.

### 4.3 Methodological quality

Overall, the methodological quality of studies was suboptimal, with all studies having at least one high/unclear risk of bias domain. This related particularly to the reference standard used, primarily due to insufficient blinding of researchers. Blinding in EHR validation studies is seldom considered, and in the common scenario when only the PPV is being assessed, blinding those interpreting the reference test is not possible (because all included patients will have been diagnosed with dementia by the index test). Nevertheless, the extent to which researchers might be influenced by knowledge of the index test result is an interesting issue and merits further discussion. The scope for misclassification of dementia status by the reference standard also led to a high risk of bias judgement in several studies, for example, by estimating the PPV of HES using the CPRD as reference standard.^27^ Furthermore, the definition of GP confirmation may differ between studies, with the potential to bias the estimates of validity reported; a reference standard that solely asks a GP whether a given patient has dementia is likely to have more misclassification than one which asks for copies of relevant tests/prescriptions to support the diagnosis.

A further two methodological limitations were not assessed by QUADAS‐2 but impacted the strength and generalisability of evidence produced by included studies. Firstly, the proportion of identified cases validated was often small, reducing the precision of the estimates of validity reported. Secondly, as identified previously,^5^ the validity estimates reported may not be generalisable to the entire database. This issue is particularly relevant to primary care databases, as not all practices take part in validation studies and data are often not available for patients who have died or have left the practice.

### 4.4 Strengths and limitations

To the best of our knowledge, this is the first review to focus specifically on the validity of dementia diagnoses in UK EHR primary care and hospitalisation databases. The comprehensiveness of the search strategy, achieved by not restricting to studies that mentioned or indexed validation terms and by an extensive bibliography and reference list search, is a major strength of this review.

A key limitation of this review is that many of the included studies did not use robust external reference diagnoses as a comparison to the codes in the EHR databases. In diagnostic test accuracy studies, it is assumed that the specificity and sensitivity of the reference test is 1, and that any misclassification is introduced by the index test alone. Clearly, this is not the case in the studies included in this review, particularly when the reference test used is another EHR database, and this fact may introduce bias into the estimates of validity reported.

The QUADAS‐2 tool used in this study is not specifically designed for the assessment of EHR validation studies. Thus, the potential for misclassifying the internal validity of a study introduced by this mismatch between study design and the risk of bias assessment tool is a limitation specific to this review. This difficulty in accurately assessing risk of bias was compounded by generally poor reporting of included studies.

An inability to assess whether publication bias is present, due to an absence of available data, is a further weakness of this review. Unlike randomised controlled trials, validation studies are often not registered. This, combined with the fact that validation often forms a small element of a larger study, may mean that researchers choose simply not to report the results of the validation exercise. As higher estimates of validity are more likely to be included in a publication, this may bias the results of this review towards overstating the validity of the codes/algorithms used to identify dementia.

## 5 CONCLUSIONS AND RECOMMENDATIONS

Through this review, we found an absence of high‐quality published literature examining the validity of different codes/algorithms for each dementia type in UK primary care and hospitalisation EHR databases. Several identified studies reported only the PPV, and a wide range of values for this measure were reported. There is therefore a need to develop, validate, and disseminate best practice codes/algorithms for identifying dementia using routine electronic health data. In addition to the PPV, future primary validation studies should calculate other measures of validity, such as sensitivity and specificity, which are more versatile and allow the code/algorithm to be used in a different study period. Similarly, future studies should aim to validate a larger number of cases to increase confidence in the estimates produced, and should make use of robust reference tests, such as an independent clinical examination based on established criteria, to reduce the potential for bias introduced by weak gold standard tests. In line with standardised reporting criteria, the codes/algorithms validated, in addition to a comprehensive description of the methods used, should be reported in the study itself or in accompanying supplementary documents if space is limited.^8^ Finally, a registry of ongoing and completed validation studies conducted in UK EHR databases would help to avoid the duplication of future work, provide a platform for the listing of “best‐practice” algorithms, and reduce the potential for publication bias.

With regards to future systematic reviews of this topic, the development and piloting of risk of bias tools specific to the validation studies of EHR diagnoses is a priority, evidenced by the limited applicability of QUADAS‐2 tool to included studies. More generally, future reviews of EHR validation studies should ensure that terms for validation are omitted from their search strategies, a recommendation consistent with previous reviews of this topic.^5^

## ETHICS STATEMENT

The authors state that no ethical approval was needed.

## CONFLICT OF INTEREST

The authors declare no conflict of interest.

## Supporting information


**Table S1.** *MEDLINE In‐process and other non‐indexed citations and MEDLINE 1946‐Present was searched using the Ovid interface and the strategy outlined below on 25/6/18*.
**Table S2**. *Overview of QUADAS‐2 signalling question judgements by study*
**Table S3.** *Justification of QUADAS‐2 signalling question judgements by study*.
