# Supplementary material for: The validity of dementia diagnoses in routinely collected electronic health records in the United Kingdom: A systematic review
Source: Pharmacoepidemiol Drug Saf. 2019 Jan 22;28(2):244–55. doi: 10.1002/pds.4669 (PMC6519035; doi:10.1002/pds.4669)
Supplement: Supplementary file 1 — Table S1. MEDLINE In‐process and other non‐indexed citations and MEDLINE 1946‐Present was searched using the Ovid interface and the strategy outlined below on 25/6/18. Table S2. Overview of QUADAS‐2 signalling question judgements by study Table S3. Justification of QUADAS‐2 signalling question judgements by study. [file PDS-28-244-s001.docx]

**Appendix 1 – Medline Search Strategy**

**Supplementary Table 1 -** *MEDLINE In-process and other non-indexed citations and MEDLINE 1946-Present was searched using the Ovid interface and the strategy outlined below on 25/6/18.*

| **No.** | **Medline Search Terms** | **Explanation of Strategy** |
| --- | --- | --- |
| 1 | CPRD.ti,ab. | Free text terms for UK specific databases |
| 2 | GPRD.ti,ab. |  |
| 3 | Clinical Practice Research Datalink.ti,ab. |  |
| 4 | General Practice Research Database.ti,ab. |  |
| 5 | The Health Improvement Network.ti,ab. |  |
| 6 | QResearch.ti,ab. |  |
| 7 | ResearchOne.ti,ab. |  |
| 8 | IMS Health.ti,ab. |  |
| 9 | Hospital Episode Statistics.ti,ab. |  |
| 10 | or/1-9 | Combined terms for UK specific databases using OR |
| 11 | dement*.ti,ab. | Free text terms for dementia |
| 12 | alzheimer*.ti,ab. |  |
| 13 | (Huntington* adj3 disease).ti,ab. |  |
| 14 | (Creutzfeldt-Jakob* adj3 disease).ti,ab. |  |
| 15 | CJD.ti,ab. |  |
| 16 | (Jakob-Creutzfeldt adj3 disease).ti,ab. |  |
| 17 | JCD.ti,ab. |  |
| 18 | (Korsakoff* adj2 syndrome).ti,ab. |  |
| 19 | ((Pick* adj3 disease) and brain).ti,ab. |  |
| 20 | (lewy* adj2 bod*).ti,ab. |  |
| 21 | or/11-20 | Combined Free texts for dementia/ Alzheimer’s using OR |
| 22 | exp Dementia/ | MeSH terms for dementia/ Alzheimer’s |
| 23 | 21 or 22 | Combined Free text and MeSH terms for dementia/ Alzheimer’s using OR |
| 24 | 10 and 23 | Combined dementia terms with UK specific database terms using AND |
| 25 | Electronic healt* record*.ti,ab. | Free text terms for routinely collected data and health maintenance organisations (less UK specific database terms) |
| 26 | Electronic medical record*.ti,ab. |  |
| 27 | Electronic clinical record*.ti,ab. |  |
| 28 | Electronic patient record*.ti,ab. |  |
| 29 | Digital healt* record*.ti,ab. |  |
| 30 | Digital medical record*.ti,ab. |  |
| 31 | Digital clinical record*.ti,ab. |  |
| 32 | Digital patient record*.ti,ab. |  |
|  |  |  |
| 33 | Computeri#ed healt* record*.ti,ab. |  |
| 34 | Computeri#ed medical record*.ti,ab. |  |
| 35 | Computeri#ed clinical record*.ti,ab. |  |
| 36 | Computeri#ed patient record*.ti,ab. |  |
| 37 | (computeri#ed adj2 record*).ti,ab. |  |
| 38 | VAMP.ti,ab. |  |
| 39 | value added medical products.ti,ab. |  |
| 40 | EHR.ti,ab. |  |
| 41 | EHRs.ti,ab. |  |
| 42 | EMR.ti,ab. |  |
| 43 | EMRs.ti,ab. |  |
| 44 | Longitudinal medical record*.ti,ab. |  |
| 45 | Longitudinal healt* record*.ti,ab. |  |
| 46 | Longitudinal clinical record*.ti,ab. |  |
| 47 | Health-care data*.ti,ab. |  |
| 48 | Healthcare data*.ti,ab. |  |
| 49 | Medical record* data*.ti,ab. |  |
| 50 | Health record* data*.ti,ab. |  |
| 51 | Clinical record* data*.ti,ab. |  |
| 52 | Longitudinal healt* data*.ti,ab. |  |
| 53 | Longitudinal clinical data*.ti,ab. |  |
| 54 | Longitudinal medical data*.ti,ab. |  |
| 55 | (Administrative adj3 data*).ti,ab. |  |
| 56 | (Automated adj3 data*).ti,ab. |  |
| 57 | (Automated adj3 record*).ti,ab. |  |
| 58 | Administrative claim*.ti,ab. |  |
| 59 | Claim* data*.ti,ab. |  |
| 60 | Primary care data*.ti,ab. |  |
| 61 | (Hospital* adj3 data*).ti,ab. |  |
| 62 | (Hospital* adj3 record*).ti,ab. |  |
| 63 | Primary care record*.ti,ab. |  |
| 64 | Routinely collected data.ti,ab. |  |
| 65 | Read cod*.ti,ab. |  |
| 66 | OXMIS cod*.ti,ab. |  |
| 67 | Clinical cod*.ti,ab. |  |
| 68 | Medical cod*.ti,ab. |  |
| 69 | ICD-10*.ti,ab. |  |
| 70 | ICD10*.ti,ab. |  |
| 71 | (Hospital* adj3 register*).ti,ab. |  |
| 72 | (population* adj3 register*).ti,ab. |  |
| 73 | or/25-72 | Combined terms for routinely collected data using OR |
| 74 | Electronic Health Records/ | MeSH terms for routinely collected data and health maintenance organisations |
| 75 | Health Records, Personal/ |  |
| 76 | Medical Records/ |  |
| 77 | Medical Records Systems, Computerized/ |  |
| 78 | Administrative Claims, Healthcare/ |  |
| 79 | Medical Record Linkage/ |  |
| 80 | Clinical Coding/ |  |
| 81 | Health Maintenance Organizations/ |  |
| 82 | or/74-81 | Combined MeSH terms for routinely collected data and health maintenance organisations using OR |
| 83 | 73 or 82 | Combined Free text and MeSH terms for routinely collected data and health maintenance organisations using OR |
| 84 | dement*.ti,ab. | Free text terms for dementia |
| 85 | alzheimer*.ti,ab. |  |
| 86 | (Huntington* adj3 disease).ti,ab. |  |
| 87 | (Creutzfeldt-Jakob* adj3 disease).ti,ab. |  |
| 88 | CJD.ti,ab. |  |
| 89 | (Jakob-Creutzfeldt adj3 disease).ti,ab. |  |
| 90 | JCD.ti,ab. |  |
| 91 | (Korsakoff* adj2 syndrome).ti,ab. |  |
| 92 | ((Pick* adj3 disease) and brain).ti,ab. |  |
| 93 | (lewy* adj2 bod*).ti,ab. |  |
| 94 | or/84-93 | Combined Free texts for dementia/ Alzheimer’s using OR |
| 95 | exp Dementia/ | MeSH terms for dementia/ Alzheimer’s |
| 96 | 94 or 95 | Combined Free text and MeSH terms for dementia/ Alzheimer’s using OR |
| 97 | 83 and 96 | Combined dementia terms with routinely collected data/ health maintenance organisation terms using AND |
| 98 | Europe*.ti,ab. | Free text terms for European Countries |
| 99 | Albania*.ti,ab. |  |
| 100 | Andorra*.ti,ab. |  |
| 101 | Armenia*.ti,ab. |  |
| 102 | Austria*.ti,ab. |  |
| 103 | Azerbaijan*.ti,ab. |  |
| 104 | (belarus* or byelarus or belorussia*).ti,ab. |  |
| 105 | Belgi*.ti,ab. |  |
| 106 | Bosnia*.ti,ab. |  |
| 107 | Bulgaria*.ti,ab. |  |
| 108 | Croatia*.ti,ab. |  |
| 109 | Cyprus.ti,ab. |  |
| 110 | Czech.ti,ab. |  |
| 111 | (Denmark or Danish).ti,ab. |  |
| 112 | Estonia*.ti,ab. |  |
| 113 | (Finland or Finnish).ti,ab. |  |
| 114 | (France or French).ti,ab. |  |
| 115 | Georgia*.ti,ab. |  |
| 116 | German*.ti,ab. |  |
| 117 | (Greece or greek).ti,ab. |  |
| 118 | Hungar*.ti,ab. |  |
| 119 | Iceland*.ti,ab. |  |
| 120 | (ireland or eire or irish).ti,ab. |  |
| 121 | Ital*.ti,ab. |  |
| 122 | Latvia*.ti,ab. |  |
| 123 | Liechtenstein*.ti,ab. |  |
| 124 | Lithuania*.ti,ab. |  |
| 125 | Luxembourg*.ti,ab. |  |
| 126 | Macedonia*.ti,ab. |  |
| 127 | (Malta or maltese).ti,ab. |  |
| 128 | Moldova*.ti,ab. |  |
| 129 | (Monaco or Monegasque).ti,ab. |  |
| 130 | Montenegr*.ti,ab. |  |
| 131 | (netherlands or holland or dutch).ti,ab. |  |
| 132 | (Norway or nowegian).ti,ab. |  |
| 133 | (Poland or polish).ti,ab. |  |
| 134 | (Portugal or portugese).ti,ab. |  |
| 135 | Romania*.ti,ab. |  |
| 136 | (Russia* or USSR or Union of Soviet Socialist Republics or Soviet Union).ti,ab. |  |
| 137 | San Marino.ti,ab. |  |
| 138 | Serbia*.ti,ab. |  |
| 139 | (slovakia* or slovak republic).ti,ab. |  |
| 140 | Slovenia*.ti,ab. |  |
| 141 | (spain or balearic islands or canary islands or spanish).ti,ab. |  |
| 142 | Swed*.ti,ab. |  |
| 143 | (Switzerland or swiss).ti,ab. |  |
| 144 | Turkey.ti,ab |  |
| 145 | (great britain or united kingdom or UK or northern ireland or scotland or channel islands or isle of man or (wales not new south wales) or (england not new england) or english or scottish).ti,ab. |  |
| 146 | Ukrain*.ti,ab. |  |
| 147 | or/98-146 | Combined Free text terms for Europe using OR |
| 148 | exp Europe/ | MeSH Term for Europe |
| 149 | 147 or 148 | Combined Free text and MeSH terms for Europe using OR |
| 150 | 97 and 149 | Combined Europe terms with dementia/EHR terms using AND |
| 151 | 24 or 150 | Combined elements of search (UK database specific element and general element) using OR |

**Appendix 2 – Supporting Material for QUADAS-2 Risk of bias assessment**

Algorithm for Domain-level judgements:

- If all signalling question are marked as “Low”, then overall => “Low”
- If any signalling question is marked as “High/Unclear”, then overall => “High”
- If all signalling question are marked as “Unclear”, then overall => “Unclear”

Signalling Question Guidance

A) STUDY DESIGN & PARTICIPANT SELECTION

1. Was a case-control design avoided?

Bias assessed: Selection bias

Low: A case-control study design with regards to the outcome of interest was not employed. This refers to a participant selection method that is dependent on true outcome status. Studies using a case-control design to assess a different outcome may be included here.

High: A case-control design for the outcome of interest was employed.

Unclear: There is insufficient information provided to make a judgement.

1. Was a consecutive or random sample of participants enrolled?

Bias assessed: Selection bias

Low: A consecutive/random sample of participants, or all eligible participants, were included.

High: A non-random sub-sample of participants were included.

Unclear: There is insufficient information provided to make a judgement.

1. Did the study avoid inappropriate exclusions?

Bias assessed: Limited challenge bias

Low: Inappropriate exclusions were avoided.

High: Inappropriate exclusions were not avoided.

Unclear: There is insufficient information provided to make a judgement.

1. Did the study have commercial funding?

Bias assessed: Sponsorship bias

Low: The study was not supported by commercial funding; the study was supported by commercial funding, BUT the separation of funders and researchers is detailed.

High: The study was supported by commercial funding.

Unclear: There is insufficient information provided to make a judgement.

B) INDEX TEST

1. Were the index test results interpreted without knowledge of the results of the reference standard?

Bias assessed: Information bias

Low: The results of the index test were interpreted without knowledge of the reference test result. This may include studies where blinding of the index test is not explicitly stated but is implied due to the sequential application of the index and reference test.

High: The results of the index test were interpreted with knowledge of the reference test result.

Unclear: There is insufficient information provided to make a judgement.

1. If a threshold was used, was it pre-specified?

The question was omitted from the quality assessment of included studies, as the concept of a threshold is not applicable to our review outcome.

C) REFERENCE STANDARD

1. Is the reference standard likely to correctly classify the target condition?

Bias assessed: Verification bias

Low: For case note review, acceptable diagnostic criteria are used (DMS-III-R, NINCDS-ADRDA) and those making assessing the outcome are appropriately qualified; for GP questionnaire, detailed questionnaire and copies of supporting documents sought; for database/linkage comparison, abroad/comprehensive range of codes used to define presence of the outcome in the reference database.

High: The reference standard is unlikely to accurately classify the target condition, due to use of no/inappropriate diagnostic criteria and unqualified assessors in case note review; insufficiently detailed GP questionnaires and lack of supporting documentation; narrow codes used to identify the outcome in the reference database.

Unclear: There is insufficient information provided to make a judgement.

1. Were the reference standard results interpreted without knowledge of the results of the index test?

Bias assessed: Information bias

Low: The results of the reference standard were interpreted without knowledge of the index test result.

High: The results of the reference standard were interpreted with knowledge of the index test result.

Unclear: There is insufficient information provided to make a judgement.

D) FLOW AND TIMING

1. Was there an appropriate interval between index test(s) and reference standard?

Bias assessed: Delayed verification bias

Low: Data for index and reference tests were collected at the same time; if retrospective, data obtained for the reference test only examined up to the date of the index test for that participant (e.g. for case note review, a participants notes are only examined up to date that dementia was coded on their entry in GPRD/HES).

High: Data examined for the reference test includes that produced after the index test.

Unclear: There is insufficient information provided to make a judgement.

1. Did all participants receive a reference standard?

Bias assessed: Partial verification bias

Low: The reference standard was performed for all (or a random sample) of the participants who received the index test.

High: The reference test was performed only on a non-random sample of the participants who received the index test; the decision to perform the reference test was based on the outcome of the index test.

Unclear: There is insufficient information provided to make a judgement.

1. Did all participants receive the same reference standard?

Bias assessed: Differential verification bias

Low: All participants received the same reference standard.

High: All participants did not receive the same reference standard; reference standards employed differed by index test outcome.

Unclear: There is insufficient information provided to make a judgement.

1. Response rate: were all participants included in the analysis?

Bias assessed: Participation bias

Low: The number of participants included in the final analysis was >70% (i.e. non-response and missing data <30%) of the total number of participants for whom data were requested for the reference test.

High: The number of participants included in the final analysis was <70% (i.e. non-response and missing data >30%) of the total number of participants for whom data were requested for the reference test.

Unclear: There is insufficient information provided to make a judgement.

**Supplementary Table 2** *– Overview of QUADAS-2 signalling question judgements by study*

| **Study** | **Study Design & Patient Selection** | | | | | **Index Test** | | **Reference Test** | | | **Flow and Timing** | | | | |
| --- | --- | --- | --- | --- | --- | --- | --- | --- | --- | --- | --- | --- | --- | --- | --- |
|  | **Q1** | **Q2** | **Q3** | **Q4** | **Domain Level** | **Q1** | **Domain Level** | **Q1** | **Q2** | **Domain Level** | **Q1** | **Q2** | **Q3** | **Q4** | **Domain Level** |
| **Brown, 2016 (GPRD)** | Low | Low | Low | Low | Low | Low | Low | Low | High | High | Low | Low | Low | Low | Low |
| **Brown, 2016**  **(GP)** | Low | Low | Low | Low | Low | Low | Low | Low | High | High | Unclear | Low | Low | Low | High |
| **Dunn, 2005 (a)** | Low | Low | Low | Low | Low | Low | Low | High | High | High | High | Low | Low | Low | High |
| **Dunn, 2005 (b)** | Low | High | Unclear | Low | High | Low | Low | Low | Unclear | High | High | Unclear | Low | Low | High |
| **Heath, 2015** | Low | High | Low | Low | High | Low | Low | Low | High | High | Unclear | Low | Low | Low | High |
| **Imfeld, 2012** | Low | Low | Low | Low | Low | Low | Low | Unclear | High | High | Unclear | Low | Low | Unclear | High |
| **Imfeld, 2013** | Low | Low | Low | Low | Low | Low | Low | Unclear | High | High | High | Low | Low | Unclear | High |
| **Ryan, 1994** | Low | Low | Low | Low | Low | Low | Low | High | High | High | Unclear | Low | Low | Low | High |
| **Seshadri, 2001** | Low | Low | Low | Low | Low | Low | Low | Low | High | High | Unclear | Low | Low | Low | High |
| **Soomerlad, 2018** | High | Low | Low | Low | High | Unclear | High | Low | Low | Low | High | Low | Low | Low | High |
| **Soo, 2014** | Low | Low | Low | Low | Low | Low | Low | High | Low | High | Low | Low | Low | Unclear | High |
| **Van Staa, 1994** | Low | Low | Low | Low | Low | Low | Low | Low | High | High | Low | Low | Low | High | High |
| **Walker, 2018**  **(HES)** | Low | Low | Low | Low | Low | Low | Low | Low | High | High | Unclear | Low | Low | Low | High |
| **Walker, 2018**  **(ONS)** | Low | Low | Low | Low | Low | Low | Low | Low | High | High | Unclear | Low | Low | Low | High |
| **Whitelaw, 1996** | Low | High | Low | Low | High | Low | Low | High | High | High | Unclear | High | Low | High | High |

**Supplementary Table 3 –** *Justification of QUADAS-2 signalling question judgements by study.*

| **Study** | | **Domain** | **Prompt** | **Justification** | | **Risk of bias** |
| --- | --- | --- | --- | --- | --- | --- |
| Brown, 2016 - validation against CPRD | | Study Design & Patient Selection | 1) Was a case-control design avoided? | Dementia status of enrolled patients was not known, as sample taken from Million Women Study cohort. | | Low |
|  |  |  | 2) Was a consecutive or random sample of participants enrolled? | All patients with a HES diagnosis of dementia were considered for validation. | | Low |
|  |  |  | 3) Did the study avoid inappropriate exclusions? | No inappropriate exclusions. See Figure 1 which details flow of patients. | | Low |
|  |  |  | 4) Did the study have commercial funding? | "This work was funded by the UK Medical Research Council (Grant No. MR/K02700X/1) and by Cancer Research UK (Grant No. C570/A11692). The funders did not influence the conduct of the study or the preparation of this report." | | Low |
|  |  | Index Test | 1) Were the index test results interpreted without knowledge of the results of the reference standard? | Blinding at this stage was inherent to the study design, as the index test (codes in HES) was applied before reference test. | | Low |
|  |  | Reference Standard | 1) Is the reference standard likely to correctly classify the target condition? | "For this study, dementia in HES records and in death certificates was defined as any of the following ICD-10 codes: E512, F00, F01, F02, F03, F10.6, F10.7, G30, or G31.0." | | Low |
|  |  |  | 2) Were the reference standard results interpreted without knowledge of the results of the index test? | As all patients assessed in GPRD were identified as cases in HES (i.e. study aims to assess only the PPV), blinding at this stage is not feasible. See Discussion for more details. | | High |
|  |  | Flow & Timing | 1) Was there an appropriate interval between index test(s) and reference standard? | As the periods of observation in CPRD and HES differ, comparison of dementia recorded in the two databases was restricted to women with overlapping observation periods | | Low |
|  |  |  | 2) Did all participants receive a reference standard? | Yes | | Low |
|  |  |  | 3) Did all participants receive the same reference standard? | Yes | | Low |
|  |  |  | 4) Response rate: were all participants included in the analysis? | All patients with a HES diagnosis of dementia and overlapping period of observation in CPRD were included. | | Low |
|  | |  |  |  | |  |
| **Study** | | **Domain** | **Prompt** | **Justification** | | **Risk of bias** |
| Brown, 2016 - validation against GP | | Study Design & Patient Selection | 1) Was a case-control design avoided? | Dementia status of enrolled patients was not known, as sample taken from Million Women Study cohort. | | Low |
|  |  |  | 2) Was a consecutive or random sample of participants enrolled? | All patients with a HES diagnosis of dementia were considered for validation. | | Low |
|  |  |  | 3) Did the study avoid inappropriate exclusions? | No inappropriate exclusions. See Figure 1 which details flow of patients. | | Low |
|  |  |  | 4) Did the study have commercial funding? | "This work was funded by the UK Medical Research Council (Grant No. MR/K02700X/1) and by Cancer Research UK (Grant No. C570/A11692). The funders did not influence the conduct of the study or the preparation of this report." | | Low |
|  |  | Index Test | 1) Were the index test results interpreted without knowledge of the results of the reference standard? | Blinding at this stage was inherent to the study design, as the index test (codes in HES) was applied before reference test. | | Low |
|  |  | Reference Standard | 1) Is the reference standard likely to correctly classify the target condition? | "For this study, dementia in HES records and in death certificates was defined as any of the following ICD-10 codes: E512, F00, F01, F02, F03, F10.6, F10.7, G30, or G31.0." | | Low |
|  |  |  | 2) Were the reference standard results interpreted without knowledge of the results of the index test? | No mention of GPs being blinded to HES case status. | | High |
|  |  | Flow & Timing | 1) Was there an appropriate interval between index test(s) and reference standard? | Insufficient information to make a judgement. | | Unclear |
|  |  |  | 2) Did all participants receive a reference standard? | Yes | | Low |
|  |  |  | 3) Did all participants receive the same reference standard? | Yes | | Low |
|  |  |  | 4) Response rate: were all participants included in the analysis? | "Informative responses were received for 73% (244/333) of the sample of women with a HES record of dementia" and "Informative replies were received for 86% (866/1004)" of patients without a HES dementia diagnosis. See Guidance. | | Low |
|  | |  |  |  | |  |
| **Study** | | **Domain** | **Prompt** | **Justification** | | **Risk of bias** |
| Dunn, 2005 (a) | | Study Design & Patient Selection | 1) Was a case-control design avoided? | True case status unknown. Patients selected from GPRD based on results of index test. | | Low |
|  |  |  | 2) Was a consecutive or random sample of participants enrolled? | "We validated a random sample of the cases and controls" | | Low |
|  |  |  | 3) Did the study avoid inappropriate exclusions? | No flow diagram presented, but all of random sample included. | | Low |
|  |  |  | 4) Did the study have commercial funding? | "This work was funded by the Wessex Medical Trust, and the Alzheimer’s Society, U.K." | | Low |
|  |  | Index Test | 1) Were the index test results interpreted without knowledge of the results of the reference standard? | Blinding at this stage was inherent to the study design, as the index test (codes in GPRD) was applied before reference test. | | Low |
|  |  | Reference Standard | 1) Is the reference standard likely to correctly classify the target condition? | "We validated. . . by writing directly to their GP for confirmation of their medical history." Insufficiently detailed GP questionnaires and lack of supporting documentation - see Guidance | | High |
|  |  |  | 2) Were the reference standard results interpreted without knowledge of the results of the index test? | No mention of blinding, though "confirmation of their medical history" infers that the GPs were made aware of the case status assigned by the index test. | | High |
|  |  | Flow & Timing | 1) Was there an appropriate interval between index test(s) and reference standard? | Patients were identified from those "on the GPRD over the age of 60 years, with data available between January 1, 1992 and January 1, 2002". Study was published in 2005, so it is likely that the GP confirmation was based on data beyond the defined study period, potentially allowing for delayed verification bias. | | High |
|  |  |  | 2) Did all participants receive a reference standard? | Yes | | Low |
|  |  |  | 3) Did all participants receive the same reference standard? | Yes | | Low |
|  |  |  | 4) Response rate: were all participants included in the analysis? | "Our validation study on 150 cases and 50 controls did not reveal any patients with incorrect diagnoses". No mention of non-response or missing data. | | Low |
|  | |  |  |  | |  |
| **Study** | | **Domain** | **Prompt** | **Justification** | | **Risk of bias** |
| Dunn, 2005 (b) | | Study Design & Patient Selection | 1) Was a case-control design avoided? | True case status unknown. Patients selected from GPRD based on results of index test. | | Low |
|  |  |  | 2) Was a consecutive or random sample of participants enrolled? | "We carried out a validation exercise of the accuracy of diagnosis using a sample of approximately . . ." No mention of how sample selected, meaning it is likely that it was not random/consecutive. | | High |
|  |  |  | 3) Did the study avoid inappropriate exclusions? | No flow diagram presented, and sample likely selected in a non-random/consecutive manner. | | Unclear |
|  |  |  | 4) Did the study have commercial funding? | "This study was funded by The Wessex Medical Trust, and by the Alzheimer’s Society, U.K. Neither of these sponsors had any involvement in the execution of the study or the decision to submit for publication." | | Low |
|  |  | Index Test | 1) Were the index test results interpreted without knowledge of the results of the reference standard? | Blinding at this stage was inherent to the study design, as the index test (codes in GPRD) was applied before reference test. | | Low |
|  |  | Reference Standard | 1) Is the reference standard likely to correctly classify the target condition? | Detailed GP questionnaire used. "We sent a questionnaire to the registered GP of each these patients, asking for details of any diagnosis of dementia, with regard to type of dementia, date of diagnosis, and confirmation of diagnosis by a specialist." | | Low |
|  |  |  | 2) Were the reference standard results interpreted without knowledge of the results of the index test? | No mention of GPs being blinded to the case status assigned by the index test. | | Unclear |
|  |  | Flow & Timing | 1) Was there an appropriate interval between index test(s) and reference standard? | Patients were identified from those "on the GPRD over the age of 60 years, with data available between January 1, 1992 and January 1, 2002". Study was published in 2005, so it is likely that the GP confirmation was based on data beyond the defined study period, potentially allowing for delayed verification bias. | | High |
|  |  |  | 2) Did all participants receive a reference standard? | It is likely based on the response to Question A2, that a non-random sample of those who received the index test also received the reference test. However, there is in sufficient information to a make a judgement. | | Unclear |
|  |  |  | 3) Did all participants receive the same reference standard? | Yes | | Low |
|  |  |  | 4) Response rate: were all participants included in the analysis? | "We obtained validation data from 95 cases (95%), and 55 (100%) controls." | | Low |
|  | |  |  |  | |  |
| **Study** | | **Domain** | **Prompt** | **Justification** | | **Risk of bias** |
| Heath, 2015 | | Study Design & Patient Selection | 1) Was a case-control design avoided? | True case status unknown. Patients selected from SPICE based on results of index test. | | Low |
|  |  |  | 2) Was a consecutive or random sample of participants enrolled? | “Prior to conducting the study, the accuracy of GP coding of young onset dementia was examined in a set of eight practices with a registered population of 51 147” – unclear how these practices were chosen, but unlikely to be random | | High |
|  |  |  | 3) Did the study avoid inappropriate exclusions? | All identified patients selected for validation, so inappropriate exclusions unlikely. | | Low |
|  |  |  | 4) Did the study have commercial funding? | “CAH was supported by National Health Service Career Research Fellowship scheme, and data set creation was supported by Scottish Government  Chief Scientist Office Applied Research Programme Grant 07/01.” | | Low |
|  |  | Index Test | 1) Were the index test results interpreted without knowledge of the results of the reference standard? | Blinding at this stage was inherent to the study design, as the index test (codes in SPICE) was applied before reference test. | | Low |
|  |  | Reference Standard | 1) Is the reference standard likely to correctly classify the target condition? | “the full primary care record including free-text and hospital letters reviewed” | | Low |
|  |  |  | 2) Were the reference standard results interpreted without knowledge of the results of the index test? | As all patients assessed by case note review were identified as cases in SPICE (i.e. study aims to assess only the PPV), blinding at this stage is difficult. Further, no mention of attempts to blind case reviewers to the results of the index test. | | High |
|  |  | Flow & Timing | 1) Was there an appropriate interval between index test(s) and reference standard? | “People with dementia were included if they were between 40 and 64 years old (inclusive) on 31 March 2007". Study was published in 2015, so it is likely that the case note review used data beyond the defined study period, potentially allowing for delayed verification bias. | | Unclear |
|  |  |  | 2) Did all participants receive a reference standard? | Yes | | Low |
|  |  |  | 3) Did all participants receive the same reference standard? | Yes | | Low |
|  |  |  | 4) Response rate: were all participants included in the analysis? | Implied as 100%. | | Low |
|  | |  |  |  | |  |
| **Study** | | **Domain** | **Prompt** | **Justification** | | **Risk of bias** |
| Imfeld, 2012 | | Study Design & Patient Selection | 1) Was a case-control design avoided? | True case status unknown. Patients selected from GPRD based on results of index test. | | Low |
|  |  |  | 2) Was a consecutive or random sample of participants enrolled? | "a random sample of 60 AD cases" | | Low |
|  |  |  | 3) Did the study avoid inappropriate exclusions? | Random sample selected for validation. | | Low |
|  |  |  | 4) Did the study have commercial funding? | "This study was funded by an unconditional grant by AstraZeneca. . . . AstraZeneca had no role in the design, methods, data collection, analysis or preparation of the manuscript." | | Low |
|  |  | Index Test | 1) Were the index test results interpreted without knowledge of the results of the reference standard? | Blinding at this stage was inherent to the study design, as the index test (codes in GPRD) was applied before reference test. | | Low |
|  |  | Reference Standard | 1) Is the reference standard likely to correctly classify the target condition? | "A questionnaire was sent to GPs . . . to obtain additional information on the clinical circumstances and the diagnostic steps taken." Insufficient detail on questionnaire reported to make a judgement. | | Unclear |
|  |  |  | 2) Were the reference standard results interpreted without knowledge of the results of the index test? | As all patients assessed by GP confirmation were identified as cases in GPRD (i.e. study aims to assess only the PPV), blinding at this stage is difficult. Further, no mention of attempts to blind GPs to the results of the index test. See Discussion for more details. | | High |
|  |  | Flow & Timing | 1) Was there an appropriate interval between index test(s) and reference standard? | Patients were identified from those "aged 65 and older who had a first-time diagnosis of AD or any unspecified dementia recorded between January 1998 and September 2008". Study was published in 2012, so it is likely that the GP confirmation was based on data beyond the defined study period, potentially allowing for delayed verification bias. | | Unclear |
|  |  |  | 2) Did all participants receive a reference standard? | Yes | | Low |
|  |  |  | 3) Did all participants receive the same reference standard? | Yes | | Low |
|  |  |  | 4) Response rate: were all participants included in the analysis? | Unclear how many validations authors attempted. No response/missing data rates represented. | | Unclear |
|  | |  |  |  | |  |
| **Study** | | **Domain** | **Prompt** | **Justification** | | **Risk of bias** |
| Imfeld, 2013 | | Study Design & Patient Selection | 1) Was a case-control design avoided? | True case status unknown. Patients selected from GPRD based on results of index test. | | Low |
|  |  |  | 2) Was a consecutive or random sample of participants enrolled? | "a random sample of 60 AD and 60 VD cases" | | Low |
|  |  |  | 3) Did the study avoid inappropriate exclusions? | Sample randomly selected, so inappropriate exclusions unlikely. | | Low |
|  |  |  | 4) Did the study have commercial funding? | "This study was supported by an unconditional grant by F. Hoffmann-La Roche Ltd." | | Low |
|  |  | Index Test | 1) Were the index test results interpreted without knowledge of the results of the reference standard? | Blinding at this stage was inherent to the study design, as the index test (codes in GPRD) was applied before reference test. | | Low |
|  |  | Reference Standard | 1) Is the reference standard likely to correctly classify the target condition? | "We sent a questionnaire to GPs . . . to obtain additional information on the clinical circumstances and the diagnostic steps taken." Insufficient detail on questionnaire reported to make a judgement. | | Unclear |
|  |  |  | 2) Were the reference standard results interpreted without knowledge of the results of the index test? | No mention of attempts to blind GPs to the results of the index test | | High |
|  |  | Flow & Timing | 1) Was there an appropriate interval between index test(s) and reference standard? | Patients were identified from those "aged 65 and older with a diagnosis of AD or VD between January 1998 and September 2008". Study was published in 2013, so it is likely that the GP confirmation was based on data beyond the defined study period, potentially allowing for delayed verification bias. | | High |
|  |  |  | 2) Did all participants receive a reference standard? | Yes | | Low |
|  |  |  | 3) Did all participants receive the same reference standard? | Yes | | Low |
|  |  |  | 4) Response rate: were all participants included in the analysis? | No mention of response rates - implied as 100% but unclear. | | Unclear |
|  | |  |  |  | |  |
| **Study** | | **Domain** | **Prompt** | **Justification** | | **Risk of bias** |
| Ryan, 1994 | | Study Design & Patient Selection | 1) Was a case-control design avoided? | True case status unknown. Patients selected from SMR based on results of index test. | | Low |
|  |  |  | 2) Was a consecutive or random sample of participants enrolled? | "Two hundred cases . . . were randomly selected" | | Low |
|  |  |  | 3) Did the study avoid inappropriate exclusions? | Sample randomly selected, so inappropriate exclusions unlikely. | | Low |
|  |  |  | 4) Did the study have commercial funding? | No commercial funding reported. | | Low |
|  |  | Index Test | 1) Were the index test results interpreted without knowledge of the results of the reference standard? | Blinding at this stage was inherent to the study design, as the index test (codes in SMR) was applied before reference test. | | Low |
|  |  | Reference Standard | 1) Is the reference standard likely to correctly classify the target condition? | Valid criteria used ("DSM-III-R"), but qualifications of case note reviewers not assessed. | | High |
|  |  |  | 2) Were the reference standard results interpreted without knowledge of the results of the index test? | No mention of attempts to blind case note reviewers to the results of the index test. | | High |
|  |  | Flow & Timing | 1) Was there an appropriate interval between index test(s) and reference standard? | Insufficient information to make a judgement. | | Unclear |
|  |  |  | 2) Did all participants receive a reference standard? | Yes | | Low |
|  |  |  | 3) Did all participants receive the same reference standard? | Yes | | Low |
|  |  |  | 4) Response rate: were all participants included in the analysis? | "One hundred and forty-six patient records (73%) were successfully traced" | | Low |
|  | |  |  |  | |  |
| Study | | Domain | Prompt | Justification | | Risk of bias |
| Seshadri, 2001 | | Study Design & Patient Selection | 1) Was a case-control design avoided? | True case status unknown. Patients selected from GPRD based on results of index test. | | Low |
|  |  |  | 2) Was a consecutive or random sample of participants enrolled? | All identified cases validated. "We identified all women who had a first-time computer diagnosis of AD . . . For each woman identified as a case, the general practitioner was sent a request . . ." | | Low |
|  |  |  | 3) Did the study avoid inappropriate exclusions? | All identified women selected for validation, so inappropriate exclusions unlikely. | | Low |
|  |  |  | 4) Did the study have commercial funding? | "This study was not specifically supported by the above grantors to the Boston Collaborative Drug Surveillance Program" | | Low |
|  |  | Index Test | 1) Were the index test results interpreted without knowledge of the results of the reference standard? | Blinding at this stage was inherent to the study design, as the index test (codes in GPRD) was applied before reference test. | | Low |
|  |  | Reference Standard | 1) Is the reference standard likely to correctly classify the target condition? | "To determine final case status, the computerized and clinical records of potential patients with AD were reviewed by 2 neurologists specializing in AD. . .based on NINCDS-ADRDA criteria" | | Low |
|  |  |  | 2) Were the reference standard results interpreted without knowledge of the results of the index test? | No mention of blinding of reviewing neurologists to index test result. | | High |
|  |  | Flow & Timing | 1) Was there an appropriate interval between index test(s) and reference standard? | Insufficient information to make a judgement. | | Unclear |
|  |  |  | 2) Did all participants receive a reference standard? | Yes | | Low |
|  |  |  | 3) Did all participants receive the same reference standard? | Yes | | Low |
|  |  |  | 4) Response rate: were all participants included in the analysis? | Data available for 81% of patients (Table 1) | | Low |
|  | |  |  |  | |  |
| Study | | Domain | Prompt | Justification | | Risk of bias |
| Soomerlad, 2018 | | Study Design & Patient Selection | 1) Was a case-control design avoided? | “True” (CRIS-defined) dementia status known. | | High |
|  |  |  | 2) Was a consecutive or random sample of participants enrolled? | All identified cases and controls validated. | | Low |
|  |  |  | 3) Did the study avoid inappropriate exclusions? | All identified cases and controls validated. | | Low |
|  |  |  | 4) Did the study have commercial funding? | “A.S. is funded by a Wellcome Trust Research Training Fellowship (200163/Z/15/Z). The data resource, G.P., and R.S. are funded by the National Institute for Health Research (NIHR) Biomedical Research Centre at South London and Maudsley NHS Foundation Trust and King’s College London.  A.S., G. Lewis and G. Livingston are supported by  the University College London Hospitals NIHR Biomedical Research Centre.” | | Low |
|  |  | Index Test | 1) Were the index test results interpreted without knowledge of the results of the reference standard? | “True” (CRIS-defined) dementia status known at time of index test, and no mention of blinding of researchers to index test result. | | Unclear |
|  |  | Reference Standard | 1) Is the reference standard likely to correctly classify the target condition? | “the CRIS database record . . . includes records from the area’s memory clinics, which are the principal U.K. dementia diagnostic services in which people are assessed by trained psychiatrists in consultation with the broader clinical team.” | | Low |
|  |  |  | 2) Were the reference standard results interpreted without knowledge of the results of the index test? | Blinding at this stage was inherent to the study design, as the reference test was applied before index test. | | Low |
|  |  | Flow & Timing | 1) Was there an appropriate interval between index test(s) and reference standard? | No. “To assess sensitivity, we examined all HES records after the CRIS dementia index date, which was the date of the first dementia diagnosis in  the CRIS database and up to March 31, 2016.” It appears that HES (index test) had access to additional time to catch dementia, and this may bias the validity estimates. | | High |
|  |  |  | 2) Did all participants receive a reference standard? | Yes | | Low |
|  |  |  | 3) Did all participants receive the same reference standard? | Yes | | Low |
|  |  |  | 4) Response rate: were all participants included in the analysis? | Implied as 100% | | Low |
|  | |  |  |  | |  |
| **Study** | | **Domain** | **Prompt** | **Justification** | | **Risk of bias** |
| Soo, 2014 | | Study Design & Patient Selection | 1) Was a case-control design avoided? | Case control on basis of CKD not dementia | | Low |
|  |  |  | 2) Was a consecutive or random sample of participants enrolled? | All patients selected for validation | | Low |
|  |  |  | 3) Did the study avoid inappropriate exclusions? | All identified patients selected for validation, so inappropriate exclusions unlikely. | | Low |
|  |  |  | 4) Did the study have commercial funding? | "This work was supported by the Chief Scientists Office for Scotland [grant number CZH/4/656]. A grant to investigate acute renal failure from Kidney Research UK in 2004 allowed the set-up of the cohort. ISD provided the SMR01 data, with NHS Grampian Health Intelligence providing an independent extract of this data to inform methodology." | | Low |
|  |  | Index Test | 1) Were the index test results interpreted without knowledge of the results of the reference standard? | Blinding at this stage was inherent to the study design, as the index test (codes in SMR) was applied before reference test. | | Low |
|  |  | Reference Standard | 1) Is the reference standard likely to correctly classify the target condition? | Experienced reviewers, but guideline/criteria used to diagnose dementia from case note data not defined. | | High |
|  |  |  | 2) Were the reference standard results interpreted without knowledge of the results of the index test? | "Clinical information had been extracted from patients’ hospital medical records by two physicians . . . blinded to the SMR01 data." | | Low |
|  |  | Flow & Timing | 1) Was there an appropriate interval between index test(s) and reference standard? | "Data were collected on selected comorbidities . . present at any time prior to, but not including any admissions at the time of the index blood sample" | | Low |
|  |  |  | 2) Did all participants receive a reference standard? | Yes | | Low |
|  |  |  | 3) Did all participants receive the same reference standard? | Yes | | Low |
|  |  |  | 4) Response rate: were all participants included in the analysis? | As study examined the validity of multiple diagnoses, availability of data on dementia not specified, but overall rate was 95% | | Unclear |
|  | |  |  |  | |  |
| **Study** | | **Domain** | **Prompt** | **Justification** | | **Risk of bias** |
| Van Staa, 1994 | | Study Design & Patient Selection | 1) Was a case-control design avoided? | Cases selected on basis of SulphonylUreas, not dementia | | Low |
|  |  |  | 2) Was a consecutive or random sample of participants enrolled? | "We validated the computer notations of 500 persons. This included all patients with a mention of a hospitalization due to hypoglycaemia or possible hypoglycaemia and a random sample of study persons with hospitalizations due to other illnesses, up to a total of 400 persons. In addition, another 100 persons without a mention of a hospitalization were randomly selected and included in the study." | | Low |
|  |  |  | 3) Did the study avoid inappropriate exclusions? | Random/consecutive samples means that inappropriate exclusions unlikely. | | Low |
|  |  |  | 4) Did the study have commercial funding? | "This research was supported by a grant from Servier AmCrique, France." | | Low |
|  |  | Index Test | 1) Were the index test results interpreted without knowledge of the results of the reference standard? | Blinding at this stage was inherent to the study design, as the index test (codes in VAMP) was applied before reference test. | | Low |
|  |  | Reference Standard | 1) Is the reference standard likely to correctly classify the target condition? | "We requested from the GPs photocopies of all discharge letters . . .The diagnoses were classified according to the ninth edition of the International Classification of Diseases" | | Low |
|  |  |  | 2) Were the reference standard results interpreted without knowledge of the results of the index test? | No mention of attempts to blind case note reviewers to the results of the index test. | | High |
|  |  | Flow & Timing | 1) Was there an appropriate interval between index test(s) and reference standard? | In SMR "We identified all persons aged 20 years or older who received, in the period between April 30, 1990 and July 3 1, 1992". For reference: "We requested from the GPs photocopies of all discharge letters covering the period from April 31, 1990 to July 1, 1992" | | Low |
|  |  |  | 2) Did all participants receive a reference standard? | Yes | | Low |
|  |  |  | 3) Did all participants receive the same reference standard? | Yes | | Low |
|  |  |  | 4) Response rate: were all participants included in the analysis? | As study examined the validity of multiple diagnoses, availability of data on dementia not specified, but overall rate was 59% | | High |
|  |  | |  | |  |  |
| Study | Domain | | Prompt | | Justification | Risk of bias |
| Walker, 2018 – comparison against HES | Study Design & Patient Selection | | 1) Was a case-control design avoided? | | True case status unknown. Patients selected from CPRD based on results of index test. | Low |
|  |  |  | 2) Was a consecutive or random sample of participants enrolled? | | All patients selected for validation | Low |
|  |  |  | 3) Did the study avoid inappropriate exclusions? | | All identified patients selected for validation, so inappropriate exclusions unlikely. | Low |
|  |  |  | 4) Did the study have commercial funding? | | “This work was supported by the Perros Trust and the Integrative Epidemiology Unit. The Integrative Epidemiology Unit is supported by the Medical Research Council and the University of Bristol (grant number MC_UU_12013/9). PGK has a professorship supported by the Sigmund Gestetner Foundation.” | Low |
|  | Index Test | | 1) Were the index test results interpreted without knowledge of the results of the reference standard? | | Blinding at this stage was inherent to the study design, as the index test (codes in CPRD) was applied before reference test. | Low |
|  | Reference Standard | | 1) Is the reference standard likely to correctly classify the target condition? | | “To use this data, we created ICD-10 code lists that correspond to the Read code lists used for the CPRD data extract.” – represents a very broad range of codes. | Low |
|  |  |  | 2) Were the reference standard results interpreted without knowledge of the results of the index test? | | No mention of attempts to blind researchers to the results of the index test. | High |
|  | Flow & Timing | | 1) Was there an appropriate interval between index test(s) and reference standard? | | Unclear whether the periods of observation in CPRD and HES differ were similar. | Unclear |
|  |  |  | 2) Did all participants receive a reference standard? | | Yes | Low |
|  |  |  | 3) Did all participants receive the same reference standard? | | Yes | Low |
|  |  |  | 4) Response rate: were all participants included in the analysis? | | Implied as 100% | Low |
|  |  | |  | |  |  |
| Study | Domain | | Prompt | | Justification | Risk of bias |
| Walker, 2018 – comparison against ONS | Study Design & Patient Selection | | 1) Was a case-control design avoided? | | True case status unknown. Patients selected from CPRD based on results of index test. | Low |
|  |  |  | 2) Was a consecutive or random sample of participants enrolled? | | All patients selected for validation | Low |
|  |  |  | 3) Did the study avoid inappropriate exclusions? | | All identified patients selected for validation, so inappropriate exclusions unlikely. | Low |
|  |  |  | 4) Did the study have commercial funding? | | “This work was supported by the Perros Trust and the Integrative Epidemiology Unit. The Integrative Epidemiology Unit is supported by the Medical Research Council and the University of Bristol (grant number MC_UU_12013/9). PGK has a professorship supported by the Sigmund Gestetner Foundation.” | Low |
|  | Index Test | | 1) Were the index test results interpreted without knowledge of the results of the reference standard? | | Blinding at this stage was inherent to the study design, as the index test (codes in CPRD) was applied before reference test. | Low |
|  | Reference Standard | | 1) Is the reference standard likely to correctly classify the target condition? | | “To use this data, we created ICD-10 code lists that correspond to the Read code lists used for the CPRD data extract.” – represents a very broad range of codes. | Low |
|  |  |  | 2) Were the reference standard results interpreted without knowledge of the results of the index test? | | No mention of attempts to blind researchers to the results of the index test. | High |
|  | Flow & Timing | | 1) Was there an appropriate interval between index test(s) and reference standard? | | Unclear whether the periods of observation in CPRD and ONS differ were similar. | Unclear |
|  |  |  | 2) Did all participants receive a reference standard? | | Yes | Low |
|  |  |  | 3) Did all participants receive the same reference standard? | | Yes | Low |
|  |  |  | 4) Response rate: were all participants included in the analysis? | | Implied as 100% | Low |
|  | |  |  |  | |  |
| **Study** | | **Domain** | **Prompt** | **Justification** | | **Risk of bias** |
| Whitelaw, 1996 | | Patient Selection | 1) Was a case-control design avoided? | True case status unknown. Patients selected from volunteering practices. | | Low |
|  |  |  | 2) Was a consecutive or random sample of participants enrolled? | Random sample of patients from volunteering practices. "Of 132 top-ranking practices (those having more than 50% of patients with a clinical Read code), 52 expressed an interest in the project and a final selection of 41 was made from these" | | High |
|  |  |  | 3) Did the study avoid inappropriate exclusions? | Random sample of patients means that inappropriate exclusions unlikely. | | Low |
|  |  |  | 4) Did the study have commercial funding? | "The project was funded by the Clinical Resource and Audit Group (CRAG) of the Scottish Office Home and Health Department." | | Low |
|  |  | Index Test | 1) Were the index test results interpreted without knowledge of the results of the reference standard? | Blinding at this stage was inherent to the study design, as the index test (codes in GPASS) was applied before reference test. | | Low |
|  |  | Reference Standard | 1) Is the reference standard likely to correctly classify the target condition? | No standard criteria and reviewers not appropriately qualified | | High |
|  |  |  | 2) Were the reference standard results interpreted without knowledge of the results of the index test? | No mention of attempts to blind case note reviewers to the results of the index test. | | High |
|  |  | Flow & Timing | 1) Was there an appropriate interval between index test(s) and reference standard? | Insufficient information to make a judgement. | | Unclear |
|  |  |  | 2) Did all participants receive a reference standard? | Random sample from volunteering practices | | High |
|  |  |  | 3) Did all participants receive the same reference standard? | Yes | | Low |
|  |  |  | 4) Response rate: were all participants included in the analysis? | Only approximately 40% (52/132) of studies expressed an interest in the study. Potential for volunteer (selection) bias. | | High |
